# Lessons from CDER’s Quality Management Maturity Pilot Programs

**DOI:** 10.1208/s12248-022-00777-z

**Published:** 2023-01-10

**Authors:** Jennifer Maguire, Adam Fisher, Djamila Harouaka, Nandini Rakala, Carla Lundi, Marcus Yambot, Alex Viehmann, Neil Stiber, Kevin Gonzalez, Lyle Canida, Lucinda Buhse, Michael Kopcha

**Affiliations:** Food and Drug Administration, Center for Drug Evaluation and Research, Office of Pharmaceutical Quality, 10903 New Hampshire Ave, Silver Spring, Maryland 20993 USA

**Keywords:** Manufacturing, Quality management, Regulation, Supply chain, Pharmaceutical quality

## Abstract

Between October 2020 and March 2022, FDA’s Center for Drug Evaluation and Research (CDER) completed two pilot programs to assess the quality management maturity (QMM) of drug manufacturing establishments. Mature quality systems promote proactive detection of vulnerabilities, prevent problems before they occur, and foster a culture that rewards process and system improvements. A CDER QMM program may help to advance supply chain resiliency and robustness and mitigate drug shortages. One pilot program evaluated seven establishments located within the U.S. that produce finished dosage form products marketed in the U.S. A second pilot program evaluated eight establishments located outside the U.S. that produce active pharmaceutical ingredients used in drug products marketed in the U.S. The execution of these pilot programs afforded FDA the opportunity to learn important lessons about the establishment QMM assessment process, scoring approach, assessor behaviors, and perceptions of the assessment questions, reports, and ratings. Many of the participating establishments reported that the QMM pilot assessments helped to identify their strengths, weaknesses, and new areas for improvement which they had not previously identified through internal audits or CGMP inspections. There has been a great deal of interest in the outcomes of CDER’s QMM pilot programs and this paper describes, for the first time, the lessons CDER learned and will continue to heed in the development of a QMM program.

**Graphical Abstract:**

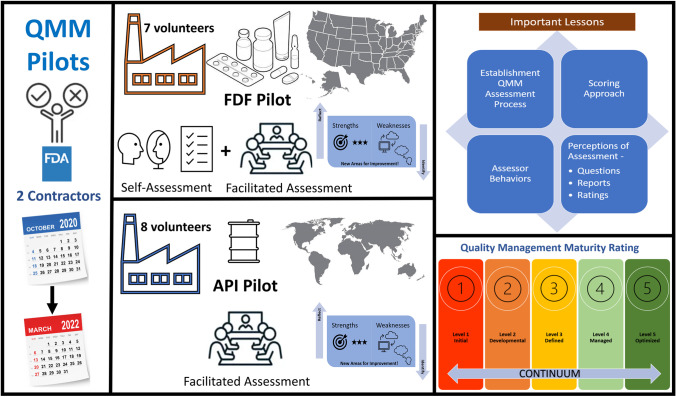

**Supplementary Information:**

The online version contains supplementary material available at 10.1208/s12248-022-00777-z.

## Introduction

Current Good Manufacturing Practice (CGMP) requirements establish systems that assure proper design, monitoring, and control of manufacturing processes and facilities, including effective quality systems. Manufacturers can exceed these standards by implementing advanced quality management practices that promote sustainable compliance and enable reliable supply chains. Quality management practices mature as companies expand their focus from meeting the standard of compliance with CGMP to a continual emphasis on proactive process and system improvements. Advanced quality systems increase proactive detection of vulnerabilities, prevent problems before they occur, and foster a culture that rewards process and system improvements. Drug manufacturers^1^ achieve higher levels of quality management maturity (QMM) when they successfully integrate business and manufacturing operations with quality practices and technological advancements to optimize product quality, enhance supply chain resiliency, mitigate drug shortages, and drive continual improvement.

Investing in mature quality management practices reduces the likelihood of disruptions or issues that lead to losses associated with poor quality and can lead to higher revenues, greater customer satisfaction, and operational efficiencies (1, 2). Over time, improvements to quality system infrastructure can enhance the robustness of a manufacturing establishment’s operations and ensure a more reliable supply of drug products (3–5). Since the mid-twentieth century, W. Edwards Deming and Joseph Juran have promoted quality concepts that form the roots of modern QMM (6). They showed that there are costs associated with poor quality (e.g., rejections, rework, unplanned repair costs, and lost sales) and demonstrated that good quality often leads to lower costs in the long term (7). Multiple years of research have demonstrated that (i) advanced quality management practices positively correlate with Pharmaceutical Quality System (PQS) effectiveness and efficiency measures, and (ii) high-performing sites, as measured by PQS effectiveness and efficiency, are more likely to implement mature behaviors across all quality practices (8). Moreover, research indicates that a high level of operational stability (i.e., the provision of capable and reliable processes and equipment) has a significant impact on delivery performance (8, 9). Recent research on the quality practices of global pharmaceutical manufacturers has found a significant positive correlation between delivery performance and the application of QMM principles associated with production (10). These findings support the hypothesis that a high degree of QMM has a positive impact on the fundamental ability to deliver supply to patients and consumers.

The United States (U.S.) Food and Drug Administration’s (FDA’s) Center for Drug Evaluation and Research (CDER) is developing a program that includes a rating system to incentivize drug manufacturers to improve QMM at their establishments. This program aims to encourage establishments to invest in a culture of quality and promote the adoption of advanced manufacturing technologies and better tools to measure overall performance. QMM assessments are not regulatory inspections and will not be used to evaluate compliance with CGMP. Rather, these assessments will be used to determine how well an establishment leverages product and process understanding at all stages of the product lifecycle, uses advanced analytics, and engages staff at all levels of the organization to drive data-driven, timely, risk-based decisions to achieve quality and business objectives.

The FDA might grant regulatory flexibility for manufacturers, including those making post-approval changes, with minimal regulatory oversight when there is greater confidence in an establishment’s commitment to quality (11). QMM ratings offer additional benefits to pharmaceutical manufacturers such as providing a means for benchmarking against industry peers and identifying areas of improvement (12). Ultimately, as establishments invest in QMM, regulators, purchasers, payors, pharmacies, healthcare providers, and patients should have increased confidence in the availability of high-quality drug products (13, 14). For more information on the benefits of a CDER QMM program, please see the 2022 white paper *Quality Management Maturity: Essential for Stable U.S. Supply Chains of Quality Pharmaceuticals* (15).

To support the development of a system to measure QMM based on objective indicators, CDER launched two pilot programs to assess producers of drug products and active pharmaceutical ingredients (APIs) used in drug products marketed in the U.S. The first pilot program evaluated seven establishments located within the U.S. that produce finished dosage form (FDF) products. The second pilot program evaluated eight establishments located outside the U.S. that produce APIs. Establishments were eligible to participate if their surveillance inspections within 5 years were classified as no action indicated (NAI) or voluntary action indicated (VAI). Participating establishments included manufacturers of prescription drug products, over-the-counter drug products, APIs, and intermediates used to make APIs.

CDER conducted these pilot programs between October 2020 and March 2022 using two contractors who were provided with a non-exhaustive list of topics to cover including supply chain management, inventory management, risk management, quality culture, and customer experience. The contractors were tasked with developing assessment protocol questions, a rubric to rate each question on an ordinal scale, and an overall QMM rating for each establishment. Contractors were selected based on their capability to recognize best practices, distinguish maturity levels using objective indicators to minimize inter-rater variability, and identify continual improvement opportunities.

The pilot program’s QMM assessments were intended to determine: (i) the level of integration of the quality system and quality objectives with business and manufacturing operations at an establishment, (ii) the agility of an establishment in responding to unexpected changes (e.g., supply chain disruptions, demand surges, deviations, natural disasters), and (iii) the resilience of an establishment’s business and production processes.

FDA staff served as pilot program observers to listen and learn from the pilot process. FDA routinely met with the contractors to reinforce the expectations for the QMM program and provided feedback to allow the contractors to fine-tune their assessment approaches. After the pilot programs concluded, both contractors gave FDA a report detailing their findings and recommendations for a future QMM program. CDER issued follow-up surveys to each of the participating establishments for feedback on the program, assessment tools and methods, and the utility of the reports. While there was no direct fee to participate in the pilot programs, follow-up surveys revealed that participants estimated their total time commitment was approximately 100 hours, though the amount of time and effort for a QMM assessment varied considerably depending on the number of staff participating in the assessment meetings.

## QMM Assessments

The QMM assessment approaches used by the two contractors were different. The use of a different contractor with a distinct strategy for each pilot increased the number of ideas generated and the potential for learning lessons related to conducting assessments. The use of different approaches limited direct comparison between the outcomes of the two pilot programs, but it did provide CDER the opportunity to analyze and implement the best aspects of each. Each assessment was conducted by at least two assessors provided by the contractor. Due to the ongoing COVID-19 public health emergency, all pilot assessments were conducted virtually.

In the FDF pilot, the assessment was performed in two stages. During the first stage, participating establishments received a self-assessment protocol with 24 questions. The participants rated their own establishments for each question using a rubric that defined five maturity levels. The second stage of the assessment was a facilitated discussion that focused on practice areas needing further clarification. These discussions provided an opportunity for the assessors to engage directly with establishment personnel and make requests for additional information and supporting documentation. The FDF pilot covered six practice areas:

1. Leadership and governance
2. Continual improvement
3. Stakeholder engagement and satisfaction
4. Knowledge management
5. Workforce engagement
6. Operations

By contrast, the API pilot assessment did not include an initial self-assessment. Rather, assessors engaged directly with staff from participating establishments, and the assessment included 66 questions. Participating establishments had the ability to upload supporting information into a portal developed by the contractor. To prepare the participants in advance of the facilitated discussions, each establishment received a document detailing what to expect during the assessment, the assessment questionnaire, an explanation of the scope of each question, and examples of supporting documentation that might be relevant. The API pilot evaluated four practice areas:

1. Sustainability
2. Risk management
3. Compliance (defined as an establishment’s approach to *exceeding* regulatory requirements and adopting best practices)
4. Quality culture

## Lessons Learned

The execution of two pilot programs afforded FDA the opportunity to learn important lessons about the establishment QMM assessment process, scoring approach, assessor behaviors, and perceptions of the assessment questions, reports, and ratings. There has been a great deal of interest in the outcomes of CDER’s QMM pilot programs and this paper describes, for the first time, the lessons CDER learned and will continue to heed in the development of a QMM program.

### Assessment Process

An early lesson regarding the assessment process was that establishments benefitted from a brief orientation ahead of the assessment. For example, it was helpful to send each establishment an introduction packet or host a pre-meeting to explain what they might expect for the assessment process and outcomes. Advance engagement was valuable because the QMM assessments are significantly different from regulatory inspections with which establishments are familiar. Establishments were provided preparatory materials at least 2 weeks in advance to allow them to adequately prepare and ensure the necessary staff and documentation were available. An establishment could request additional preparatory time if needed. The establishments found it helpful to have concrete examples of the types of documents that could substantiate the QMM ratings. Assessors and participants found value in using the self-assessments as they gave a voice to the establishments in the assessment process. The information gathered from the self-assessments helped direct the facilitated discussions to areas that needed further elaboration or clarification.

Through the pilot programs, CDER learned that some questions are best answered by corporate leaders who have responsibilities across multiple establishments, when relevant, and other questions are best answered by establishment personnel. For this reason, elements within the practice areas need to be grouped appropriately to enable participants to plan to have the necessary staff available when their input is needed. The assessments were more efficient when the appropriate staff were available to address relevant practice areas, further emphasizing the benefit of providing assessment questions ahead of time. Because of the ongoing public health emergency, assessors were unable to perform independent on-site interviews with management and staff. Independently interviewing different levels of staff within the organization may be important to gauge the culture at an establishment.

CDER’s observers noted that applying a strict time limit to each question was not practical. Both pilot programs initially assigned a 15-min time limit for each question, but some questions could not effectively be covered in that timeframe. Some conversations were rushed and did not yield the information needed to evaluate a specific practice area. Furthermore, some elements, such as quality risk management, knowledge management, innovation, and continual improvement were covered by questions in multiple practice areas. There will be a need to streamline the questions and framework to avoid overlap or redundancy. Compound questions that covered multiple aspects of a particular topic within a practice area posed a challenge to some participants. Furthermore, some questions were leading or did not give participants an opportunity to elaborate in their responses.

It proved beneficial to provide examples of the types of documentation needed to substantiate an establishment’s response to an assessment question. It will be important to ensure that practice areas and the answers to assessment questions are adequately supported with documentation to substantiate self-assessment, facilitated assessment, and maturity level determinations.

### Scoring Approach, Communications, and Assessor Behaviors

**Fig. 1:**
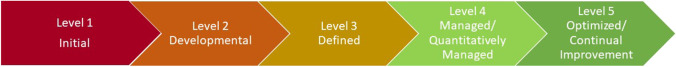
Levels of maturity. The names for scoring levels 4 and 5 differed between the pilots. Both are included

To objectively assess an establishment’s QMM, assessors used a scoring approach to determine the level of maturity based on an ordinal scale of levels one through five (Fig. 1). Assessors assigned scores to each practice area and determined a final aggregated score for each establishment. When scores differed among assessors, the team employed a consensus process to align on the final score and resolve any differences. A systematic and objective approach for managing conflicting scores will be needed.

In some cases, assessors encountered challenges when using the scoring rubric. Unnecessary complexity in the level descriptions and the rubric may lead to subjectivity in scoring. Redundant topics within or between practice areas may reduce the accuracy of scoring if these topics are accounted for more than once. A simplified rubric would increase the likelihood that the scores accurately reflect the maturity of each unique practice area. FDA will need to develop objective criteria to discern between the maturity levels and to score QMM assessments in a consistent, unbiased, data-driven, and scientific manner. The scoring system will also need to account for missing and outlier data. For example, some questions in the assessment were not applicable to all establishments, and some establishments could not or did not answer a question. Multiple assessors may be needed per assessment to minimize bias and maximize inter-rater reliability. A standardized scoring approach will allow establishments to benchmark against peers and track their own progress over time.

FDA will need to train assessors on the assessment tool, scoring rubric, CGMP regulations, and FDA’s quality guidance documents so that they can distinguish between behaviors that meet or exceed CGMP expectations. Although QMM assessments are not regulatory inspections, establishment personnel may be apprehensive when engaging with the assessors. Therefore, FDA will also need to train assessors in interview techniques to effectively engage staff at the establishment and efficiently manage time. Establishment personnel will need sufficient time to understand a question and respond. In the pilot programs, assessors did not always adhere to the planned assessment questions and sometimes missed opportunities to ask follow-up questions. Assessors will need to avoid asking leading or yes/no questions when an open-ended question would better serve the assessment. During the facilitated discussions, assessors need to remain neutral and avoid opining or asking questions out of intellectual curiosity that are not directly relevant to the QMM assessment. Assessors also need to avoid the use of jargon and should explain questions and instructions using plain language.

**Fig. 2:**
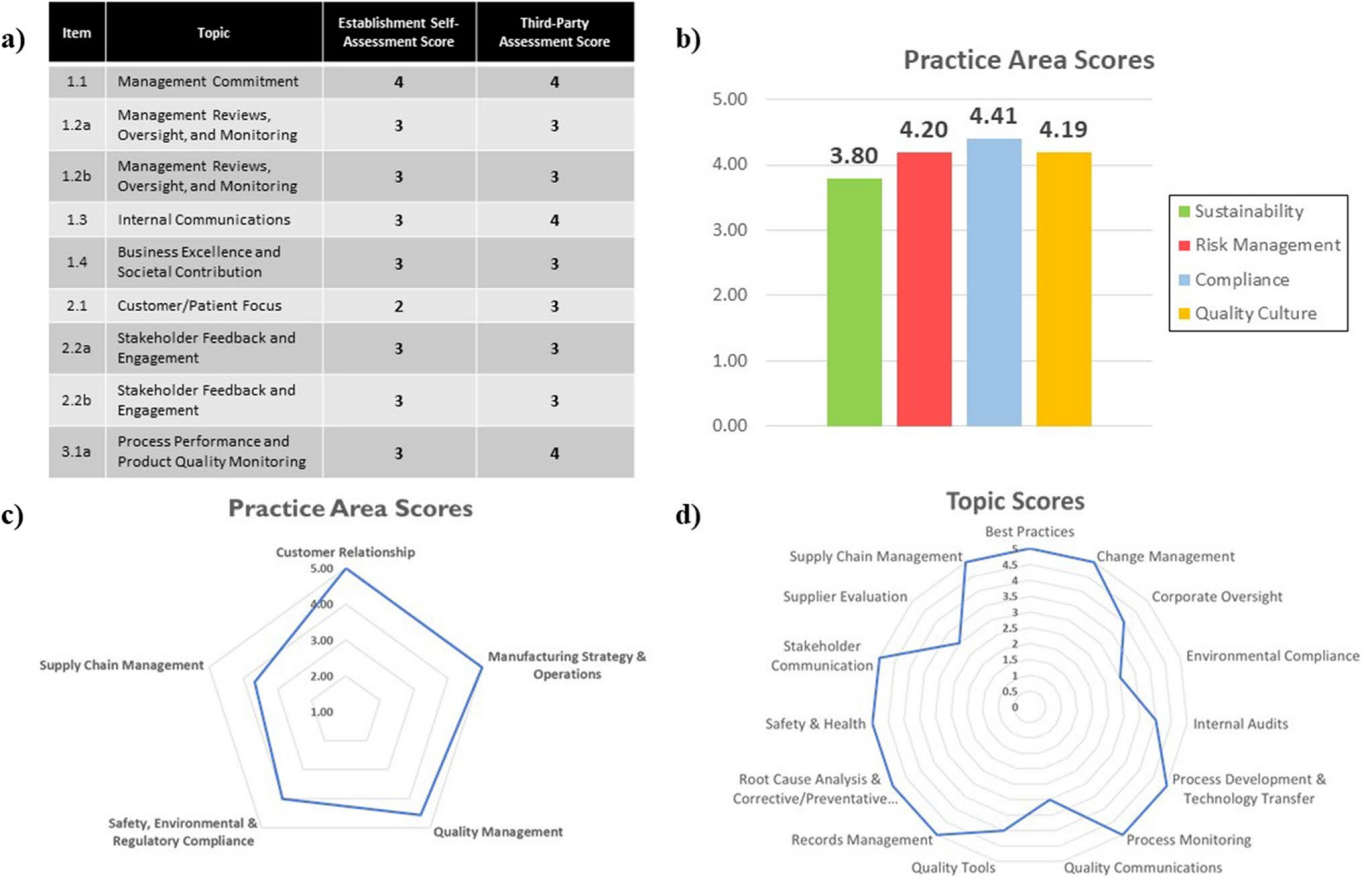
Examples of Pilot Assessment Communications. **a** Sample from a domestic establishment report showing tabulated results for the self-assessment and third-party assessment. Attributes are topics within a practice area. **b** Sample from a foreign establishment report showing the scores for each practice area. **c** Sample from a foreign establishment report showing the maturity level of topics within a practice area. **d** Sample from a foreign establishment report showing the maturity level for each sub-topic within a practice area

The assessment reports provided to FDF establishments included how participants scored themselves (Fig. 2a) and how assessors scored the establishment in each practice area (Fig. 2b). In addition, each establishment received an overall QMM score and a final report containing commentary on each practice area as well as recommendations for continual improvement. Assessors, participants, and the FDA generally preferred data presented visually in radar/spider plots rather than in tabulated scores, as establishments could more readily visualize their performance in different practice areas (Fig. 2c, d).

### Feedback and Stakeholder Engagement

CDER has received feedback on the development of a QMM program in several ways, including from the pilot program participants, from stakeholders at the 2020 Duke-Margolis workshop *Understanding How the Public Perceives and Values Pharmaceutical Quality* (16), stakeholder meetings, and the 2-day 2022 CDER QMM Workshop (17). Some of the key considerations and elements of a QMM program described in the white paper *Quality Management Maturity: Essential for Stable U.S. Supply Chains of Quality Pharmaceuticals* stemmed from this feedback. The overall sentiment on the QMM program has been positive. For example, 99% of over 400 stakeholders polled at the 2022 CDER QMM Workshop believed that purchasers of drug products or APIs should “consider the QMM of the facility that manufactures them.”

Most of the feedback from the pilot program participants was positive. Many participants reported that the QMM assessments helped to identify their strengths, weaknesses, and new areas for improvement that they had not previously identified through internal audits or CGMP inspections (Table I).

**Table I:** Examples of Feedback Received from Participants of the QMM Pilot Programs

On the use of QMM assessments for internal improvement	On the potential use of supplier QMM scores for supplier evaluation
“The assessment results will be used to 1) celebrate the practice areas where the establishment scored well, 2) drive continuous improvement by identifying improvement opportunities, 3) use the results to influence internal… initiatives.”	“QMM data from suppliers would be very helpful. Often suppliers are half a world away and the QMM scores and assessment narrative would provide valuable insights into the establishment’s quality culture.”
“We may review these results when developing goals and objectives, and for strategic planning.”	“This will be an input into the supplier qualification and management program for the supplier. We will have an indication of the quality maturity at the firm based on the results.”
“The assessment results were shared with the relevant business/system owners. This ‘outside’ perspective is always a welcome look into what we can do better and learn from others.”	“In the same way that QMM data is useful in assessing a potential API supplier, the data could be useful in assessing a contract lab or other contract facility.”
“Our establishment will use the assessment results to target improvement areas in the Quality Plan.”	“We could envisage using the QMM score to reduce the frequency, content, and time spent on vendor audits.”
“The assessment results will be used for the improvement of processes and programs and for communication within the corporate organization.”	“QMM scores from API suppliers can be used as part of supplier selection.”
“We will look at the various behaviors/actions listed in each maturity level and strive to move to the next level.”	“QMM scores from API suppliers would most likely be taken into account as part of the overall evaluation of a supplier during the selection or ongoing supplier maintenance evaluation program.”

FDA will consider the overall feedback from the pilot programs and stakeholder outreach to make operational decisions for the QMM program. An example of an operational decision is whether the QMM assessments would be best executed by FDA personnel, a contracted third party, or combination of both. If the FDA were to conduct the assessments, CDER would have to hire, onboard, and train additional staff. If a third party performs the assessments, this will require extensive training and certification of assessors. Operationalizing a QMM program will require substantial resources. The implementation of this program and its rate of scale-up depends on a number of factors, including funding.

It also remains to be determined if assessments are best executed virtually, on-site, or using a hybrid approach. Some pilot participants favored the virtual model as the most efficient use of their time since they could schedule subject matter experts when needed. However, assessors may benefit from having some on-site engagement with the establishments to better gauge the culture and interview staff and leadership independently. To move the development of a QMM program forward, CDER will continue to work with stakeholders to address these and other decisions through engagements with trade associations, public stakeholders, and advisors. An FDA Advisory Committee Meeting in November 2022 concluded with a unanimous vote (9–0) in favor of establishing a CDER QMM program.

Some stakeholders have voiced confusion about the nomenclature of the program and the ratings. For example, some stakeholders have confused FDA’s Quality Metrics (QM) Program with QMM. The Quality Metrics Program is separate from the QMM pilot program. Quality Metrics are an objective way to measure, evaluate, and monitor the product and process lifecycle (18). Some have confused the term “maturity” with the age of a manufacturing facility. Other stakeholders have mistakenly assumed that QMM ratings are product quality ratings instead of ratings of establishments. It is important to note that a QMM assessment *will not* evaluate product quality. Rather, QMM assessments evaluate an establishment’s culture, behaviors, attitudes, and enhanced quality management practices. Establishments with high QMM are more likely to produce a robust supply and experience fewer quality-related disruptions.

Stakeholders have inquired about how long a QMM rating will last. FDA is determining whether other quality indicators of an establishment’s operations (e.g., Quality Metrics) will be used in addition to the QMM assessment to support confidence in the rating (19). These factors may impact the longevity of the rating and the frequency of QMM assessments.

**Fig. 3:**
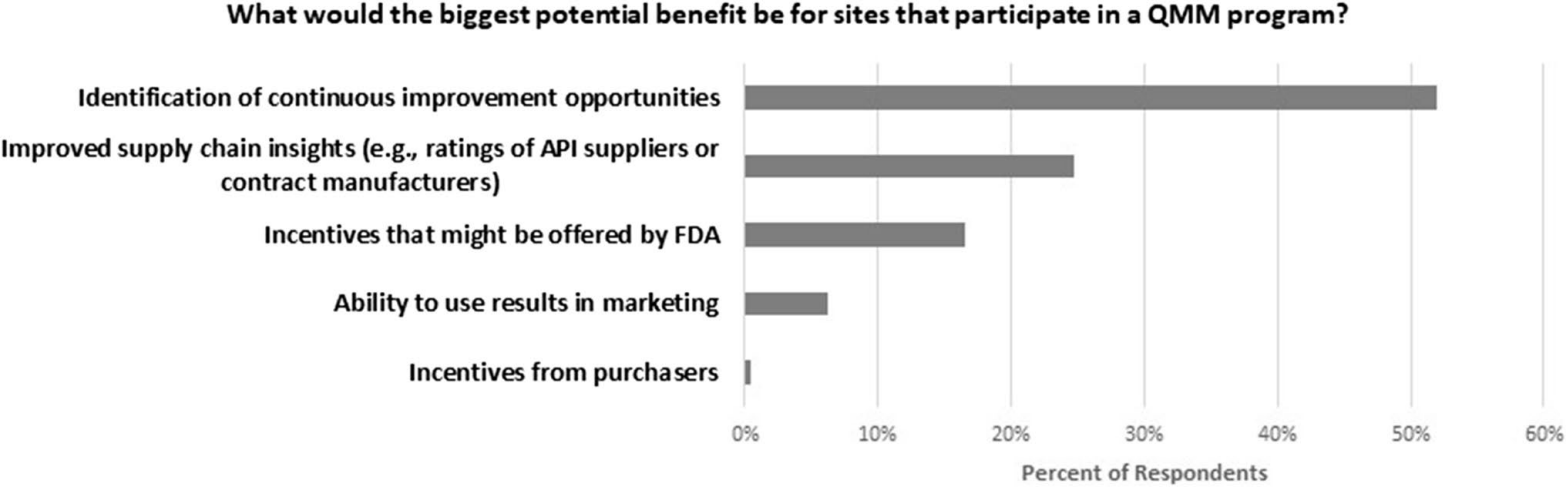
2022 CDER QMM Workshop Poll Results. 223 respondents to a poll of CDER workshop participants

There has been much interest surrounding the potential incentives that might promote voluntary participation in a future CDER QMM program. Separate from the pilot programs, CDER’s 2-day QMM Workshop provided opportunities for stakeholder engagement. An audience poll of over 200 stakeholders at the 2022 CDER QMM Workshop asked, “What would the biggest potential benefit be for sites that participate in a QMM program?” (Fig. 3). Respondents believed that the biggest perceived benefits were not regulatory incentives (17%). Rather, the biggest perceived benefits were the identification of continuous improvement opportunities (52%) and improved supply chain insight (25%) (e.g., using ratings of API suppliers or contract manufacturers). CDER will continue to weigh the risks, benefits, and potential incentives of a QMM program. Implementation of the QMM program will require careful design and education for all impacted stakeholders.

## Conclusion

Executing two QMM pilots provided FDA with insights into how to design and implement a future QMM assessment protocol. The program will continue to be developed with stakeholder input. CDER is committed to providing stakeholders with additional information on the QMM program as it develops, including the implementation timeline, how ratings will be determined, and the measures of program success that track year-to-year progress on a continual improvement journey.

A QMM program may enhance supply chain resiliency and robustness and mitigate drug shortages. All participating establishments should benefit from the identification of targeted areas for continual improvement. The overall supply chain should also benefit as manufacturers with higher QMM focus on continual improvement and are therefore more likely to embrace advanced manufacturing technologies that enhance the capability, robustness, and commitment to quality by the pharmaceutical industry (20). The potential benefits of a QMM program are clear: manufacturers with higher QMM get recognition in the market, purchasers and payors get more insight and confidence into the supply chain of the drugs they buy or reimburse, and patients, pharmacies, and healthcare professionals get improved availability, particularly of drugs at risk of shortage. Everyone will have more confidence in their next dose of medicine.

## Supplementary Information

ESM 1 (DOCX 15.9 kb)

## Data Availability

All data generated or analysed during this study are included in this published article and its supplementary information files.

## Footnotes

1 Manufacture, processing, packing, or holding of a drug product includes packaging and labeling operations, testing, and quality control of drug products (21 CFR 210.3(b)(12)).
