# Supplementary material for: Lessons from CDER’s Quality Management Maturity Pilot Programs
Source: AAPS J. 2023 Jan 10;25(1):14. doi: 10.1208/s12248-022-00777-z (PMC9831683; doi:10.1208/s12248-022-00777-z)
Supplement: Supplementary file 1 — (DOCX 15.9 kb) [file 12248_2022_777_MOESM1_ESM.docx]

**Supplementary Table 1: Sample from a foreign establishment report showing the scores for each practice area**

| **Pillar** | **Benchmarking Average** |
| --- | --- |
| Sustainability | 3.80 |
| Risk Management | 4.20 |
| Compliance | 4.41 |
| Quality Culture | 4.19 |
| **Overall QMM Score** | **4.15** |

**Supplementary Table 2: Sample from a foreign establishment report showing the maturity level of topics within a practice area**

| **Topics** | **Average of Rating** |
| --- | --- |
| Customer Relationship | 5.00 |
| Manufacturing Strategy & Operations | 5.00 |
| Quality Management | 4.56 |
| Safety, Environmental & Regulatory Compliance | 4.00 |
| Supply Chain Management | 3.67 |

**Supplementary Table 3: Sample from a foreign establishment report showing the maturity level for each sub-topic within a practice area**

| **Sub-Topics** | **Average of Rating** |
| --- | --- |
| Best Practices | 5.00 |
| Change Management | 5.00 |
| Corporate Oversight | 4.00 |
| Environmental Compliance | 3.00 |
| Internal Audits | 4.00 |
| Process Development & Technology Transfer | 5.00 |
| Process Monitoring | 5.00 |
| Quality Communications | 3.00 |
| Quality Tools | 4.00 |
| Records Management | 5.00 |
| Root Cause Analysis & Corrective/Preventative Actions | 5.00 |
| Safety & Health | 5.00 |
| Stakeholder Communication | 5.00 |
| Supplier Evaluation | 3.00 |
| Supply Chain Management | 5.00 |

**Supplementary Table 4: 2022 CDER QMM Workshop Poll Results. 223 respondents to a poll of CDER workshop participants**

**Poll Question:** What would be the biggest potential benefit for sites that participate in a QMM program?

| **Potential Benefit** | **Number of Respondents** | **Percentage of Respondents** |
| --- | --- | --- |
| Identification of continuous improvement opportunities | 116 | 52.02% |
| Improved supply chain insights (e.g., ratings of API suppliers or contract manufacturers) | 55 | 24.66% |
| Incentives that might be offered by FDA | 37 | 16.59% |
| Ability to use results in marketing | 14 | 6.28% |
| Incentives from purchasers | 1 | 0.45% |
